# Weight status and hypertension among adolescent girls in Argentina and Norway: Data from the ENNyS and HUNT studies

**DOI:** 10.1186/1471-2458-9-398

**Published:** 2009-10-30

**Authors:** Marit Stray-Pedersen, Ragnhild M Helsing, Luz Gibbons, Gabriela Cormick, Turid L Holmen, Torstein Vik, José M Belizán

**Affiliations:** 1 Department of Laboratory Medicine, Children's and Women's Health, Faculty of Medicine, Norwegian University of Science and Technology, Trondheim, Norway; 2 Department of Mother & Child Health Research, Institute for Clinical Effectiveness and Health Policy (IECS), Buenos Aires, Argentina; 3 HUNT Research Center, Department of Public Health and General Practice, Faculty of Medicine, Norwegian University of Science and Technology, Verdal, Norway

## Abstract

**Background:**

To provide data on overweight, obesity and hypertension among adolescent girls in Norway and Argentina.

**Methods:**

Data was obtained from two population-based, cross-sectional and descriptive studies containing anthropometric and blood pressure measurements of 15 to 18 year old girls. The study included 2,156 adolescent girls from Norway evaluated between 1995 and 1997, and 669 from Argentina evaluated between 2004 and 2005.

**Results:**

Around 15% of adolescent girls in Norway and 19% in Argentina are overweight or obese. Body mass index (BMI) distribution in these two countries is similar, with a low percentage (< 1%) of girls classified as thin. Norwegian adolescents show a height mean value 8 cm taller than the Argentinean. Obesity is strongly associated with systolic hypertension in both populations, with odds ratios of 11.4 [1.6; 82.0] and 28.3 [11.8; 67.7] in Argentina and Norway, respectively. No direct association between BMI and systolic hypertension was found, and only extreme BMI values (above 80^th ^- 90^th ^percentile) were associated with hypertension.

**Conclusion:**

This study confirms a current world health problem by showing the high prevalence of obesity in adolescents and its association with hypertension in two different countries (one developed and one in transition).

## Background

Overweight, obesity and hypertension are significant risk factors for increased morbidity and mortality, not only in high-income but also in medium and low-income countries, including countries in Latin America [1,2]. The prevalence of overweight and obesity in children has shown a remarkable increase over the last decades [3]. An increase in the prevalence of hypertension has also been reported, although to a lesser extent [4].

Overweight and obesity, as well as elevated blood pressure in childhood and adolescence tend to track into adult life [5]. Thus, assessing the prevalence of overweight, obesity and hypertension in children and adolescents may predict the future health of a country's population [6].

The aim of this study was to provide data on overweight, obesity and hypertension of adolescent girls in a middle-income country (Argentina) and a high-income country (Norway). Two already existing databases from these two countries were analysed in order to supply information about a world-wide recognised health problem.

## Methods

### Study design and population

This study analyses data from two population-based, cross-sectional and descriptive studies which included anthropometric and blood pressure data of girls aged between 15 and 18.

The Norwegian study, Young-HUNT 1, is the youth part of The Health Study of Nord-Trøndelag carried out between 1995 and 1997 (HUNT 2) [7]. Data was collected by HUNT Research Center, at the time part of the National Institute of Public Health. The Argentinean study employed is the National Survey of Nutrition and Health (ENNyS), carried out between 2004 and 2005 by the Ministry of Health in Argentina [8].

The Young-HUNT study included all adolescents in the county of Nord-Trøndelag. In this county, higher education prevalence and average income are slightly lower than the average in Norway, while it is close to the average when it comes to geography, economy, industry, sources of income, age distribution, morbidity and mortality [7]. Participants in the ENNyS were selected using a probabilistic complex sample design including different socioeconomic levels from both large and small cities of all provinces of Argentina. The survey was designed in order to make it possible to extrapolate the results to the whole population of Argentina [9].

In the present study, only adolescent girls aged 15 to less than 18 years were included; 2,156 from Young-HUNT and 676 from the ENNyS. The Young-HUNT involved only adolescent population and the ENNyS involved women in reproductive age.

The total number of girls in the 15 to under 18 age group living in Nord-Trøndelag in 1995 was 2,487, implying that our study population involves 86.7% of the total population [10].

Of the 2,156 adolescents girls that completed the questionnaire and accepted to participate in the HUNT study, 2,050 (95%) were included in the analysis. Exclusions were due to missing data on height, weight, diastolic or systolic blood pressure in the Norway database.

ENNyS included women from 89 small and large localities of the country, while those living in collective homes (orphanages, religious institutions, hospitals) were excluded [8]. The ENNyS was designed to include 1,200 women from each of Argentina's six regions, 7,200 in total. Among these, 676 were in the 15 to under 18 age group, and of those 669 (99%) were included in the analysis. Exclusions were due to missing diastolic or systolic blood pressure data on the Argentinean database.

### Outcome measures

In the HUNT study, weight, height and blood pressure were measured by trained nurses travelling to the adolescents' schools. Weight and height were measured using meter measures and weight scales that were internally standardized. The subjects wore light clothes (T-shirts and trousers) and no shoes. Blood pressure was measured with the subject seated and relaxed and the arm resting on a table at the same height level as their heart. An automatic oscillometric method was used (507 N monitor, Criticare System Inc.), upon inflation technique, with an appropriate cuff according to the arm circumference. Blood pressure was measured after two minutes of rest, and three consecutive measurements were then taken with intervals of one minute. For the analysis, the mean of the two last measurements were used.

In the ENNys study, weight, height and blood pressure were measured during home visits by standardized surveyors. Weight was measured on portable mechanical scales, with the study subject wearing no clothes, or a minimum of clothes. Height was measured with the subject standing barefoot, using a portable aluminum meter, and gently pulling the mastoid apophysis upwards, with the Frankfurt plane in a horizontal position. Consecutive measurements were taken until the difference between two measurements reached three mm or less, and the last measurement was used for the analysis. Arterial blood pressure was measured according to the WHO Hypertension Expert Committee norms [11]. Measurements were carried out in standardized conditions, using a mercury sphygmomanometer with a scale graduated in 2 mmHg. Two measurements were taken with 5 minute intervals. For analysis, the mean of the two measurements was used.

Body mass index (BMI) was calculated as body weight (kg) divided by the squared value of height (m). Overweight, obesity and thinness were defined using the International age and sex specific BMI cut-off values for adolescents proposed by Cole et al [12,13]. Cole's cut-off points were developed using centiles related to BMI 17, 25 and 30 kg/m2 at age 18. These standards are based on averaging across a heterogeneous population including data from 6 different countries, one of them in South America.

Hypertension and pre-hypertension were defined using as reference the standards of the National High Blood Pressure Education Program Working Group on High Blood Pressure in Children and Adolescents [14]. These standards define the blood pressure for each individual as the value calculated from the average of three different measurements. In this study, the mean of two measurements was used, since a third measurement was not available.

• Hypertension was defined as values of SBP and/or diastolic BP (DBP) that are ≥ 95th percentile for gender, age, and height.

• Prehypertension was defined as values of SBP or DBP levels that are ≥ 90th percentile but < 95th percentile for gender, age, and height.

### Statistical analysis

The study population's characteristics were reported with means and standard deviations stratified by country. Proportions with their respective confidence interval were calculated in order to describe the distribution of weight status and blood pressure.

The association between systolic pre-hypertension and hypertension, and diastolic pre-hypertension and hypertension with weight status categorised in three different groups (normal weight, overweight and obesity) was studied using a logistic regression model stratified by country. The normal weight group was used as the reference for the analysis of each country. We reported the odds ratio with the respective confidence interval for each association.

**Figure 1:**
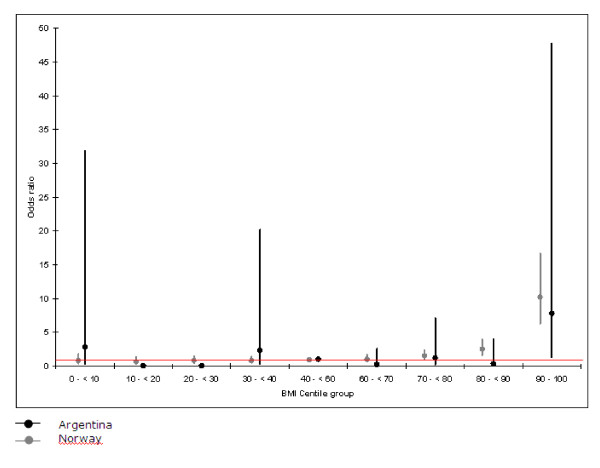
**Odds ratio for being hypertensive as opposed to normotensive according to Body Mass Index (BMI) centile group**.

BMI was categorized by deciles and its association with systolic hypertension was estimated by odds ratio with their respective confidence interval; 40-60 deciles were considered as the reference group and the analysis was carried out separetely for each country. The results were reported in a figure 1. All centiles and z-scores were calculated according to CDC growth charts from year 2000 using Epi-Info (3.4.3).

All the analyses performed with the ENNyS database were adjusted for the complex sample design weight. For the HUNT database, standard analysis was used.

The analyses were performed using SPSS version 13.

### Ethics

Ethical approval was obtained from the Regional Committee of Ethics and Norwegian Data Inspectorate Board and from the Ethical Committee in Hospital de Clínicas of the University of Buenos Aires. The authorization to analyze the HUNT data was obtained through the Norwegian University of Science and Technology and the authorization to analyze the ENNyS data through the Institute for Clinical Effectiveness & Health Policy.

In Norway, all participants, and parents of those younger than 16 years, had to sign an informed consent in order to participate in the study. In Argentina, the "head of family" or another responsible adult signed an informed consent for the adolescents to participate in the study.

## Results

The characteristics of the two populations are shown in Table 1. The mean age was similar in both populations. The Norwegian girls were taller and heavier than the Argentine girls, but the BMI was similar. The Norwegian girls had a higher mean systolic blood pressure, whereas the Argentinean girls had a higher mean diastolic blood pressure.

**Table 1:** Descriptive statistics of the study population(s), girls aged between 15 and 18 years from Argentina (2004 and 2005) and Norway (1995-1997)

	**Argentina** **(ENNyS)** **(n = 669)**		**Norway** **(Young-HUNT)** **(n = 2,050)**	
	Mean **	**SD**	**Mean**	**SD**
Age (yrs)	16.5	0.85	16.4	0.88
Height (cm)	158.4	6.89	166.4	6.00
Weight (kg)	55.2	9.67	60.3	9.44
BMI (kg/m^2^)	22.0	3.75	21.8	3.13
Mean systolic blood pressure (mmHg)	107.1	10.79	119.9	10.48
Mean diastolic blood pressure (mmHg)	67.0	9.83	64.0	7.47

** calculated by SPSS complex analysis

The distribution of weight status was similar in the two populations (Table 2). Very few adolescent girls, less than 1%, from both samples were classified as thin. 14.5% were classified as overweight in Argentina and 12.1% in Norway. The proportion of obesity was 4.3% and 2.7% respectively.

**Table 2:** Distribution of BMI/Percentage of thinness, normal weight, overweight and obesity in Argentina (2004 and 2005) and Norway (1995-1997) according to Cole (2000, 2007)

	**Argentina** **(n = 669)**	**Norway** **(n = 2,050)**
	
	% [CI_95%_]**	**% [CI_95%_]**
Thinness	0.6 (0.3; 1.3)	0.9 (0.5; 1.3)
Normal weight	80.6 (74.6; 85.4)	84.2 (82.7; 85.8)
Overweight	14.5 (10.2; 20.3)	12.1 (10.7; 13.6)
Obesity	4.3 (2.1; 8.5)	2.7 (2.0; 3.4)

**95% CI calculated by SPSS complex analysis

Norwegian girls had a prevalence of 29.1% of systolic pre-hypertension and 16.6% of hypertension and Argentinean girls had 7.0 and 3.5% respectively. The prevalence of diastolic pre-hypertension and hypertension was 1.3 and 0.4% in the Norwegian group and 9.0 and 3.8% in the Argentinean one (Table 3).

**Table 3:** Prevalence of systolic and diastolic low/normal blood pressure, prehypertension and hypertension in Argentina (2004 and 2005) and Norway (1995-1997) according to the National High Blood Pressure Education Program Working Group on High Blood Pressure in Children and Adolescents (2004)

	**Systolic**		**Diastolic**	
	
	**Argentina** **(n = 669)**	**Norway** **(n = 2,050)**	**Argentina** **(n = 669)**	**Norway** **(n = 2,050)**
	
	% [CI_95%_]**	**% [CI_95%_]**	% [CI_95%_]**	**% [CI_95%_]**
Low/normal blood pressure	89.5 (85.2; 92.7)	54.3 (52.2; 56.5)	87.2 (82.2; 90.9)	98.2 (97.6; 98.8)
Pre hypertension	7.0 (4.7; 10.4)	29.1 (27.1; 31.1)	9.0 (5.8; 13.6)	1.3 (0.9; 1.9)
Hypertension	3.5 (1.7; 7.0)	16.6 (15.0; 18.2)	3.8 (2.2; 6.7)	0.4 (0.2; 0.7)

**95% CI calculated by SPSS complex analysis

Table 4 shows overweight to be strongly associated with systolic hypertension in Norway whereas obesity is associated with hypertension in both countries. A lower but statistically significant association between obesity and diastolic association is also seen in both countries.

**Table 4:** Odds ratio for high systolic or diastolic blood pressure when overweight or obese compared to normal weight (Argentina n = 669, Norway n = 2,050)

		**Systolic**				**Diastolic**			
		
		**Prehypertension**		**Hypertension**		**Prehypertension**		**Hypertension**	
		
		**OR**	**CI_95%_**	**OR**	**CI_95%_**	**OR**	**CI_95%_**	**OR**	**CI_95%_**
Argentina**	Normal weight (n = 561)	1.0	-	1.0	-	1.0	-	1.0	-
	Overweight (n = 80)	0.3	[0.1; 0.8]	3.3	[0.5; 21.5]	0.5	[0.2; 1.5]	4.5	[1.2; 17.3]
	Obesity (n = 28)	0.9	[0.2; 4.1]	11.4	[1.6; 82.0]	0.3	[0.05; 2.6]	2.2	[0.4; 10.8]

Norway	Normal weight (n = 1,745)	1.0	-	1.0	-	1.0	-	1.0	-
	Overweight (n = 249)	2.2	[1.6; 3.0]	3.8	[2.7; 5.4]	1.2	[0.4; 4.3]	1.0	[0.1; 8.2]
	Obesity (n = 56)	4.2	[1.5; 11.1]	28.3	[11.8; 67.7]	14.8	[5.8; 37.3]	5.1	[0.6; 42.4]

** calculated using SPSS complex analysis

Associations of BMI grouped by deciles with hypertension in the two countries are shown in figure 1. It may be seen that hypertension was associated with BMI only in the upper extreme values, above the 80^th ^percentile in Norway and above the 90^th ^percentile in Argentina.

## Discussion

This study confirms in two different countries (one developed and one in transition) a current health problem in the world, namely the high prevalence of obesity in adolescents and its association with hypertension. Systolic hypertension among obese Norwegian adolescent girls was 28.3 times the odds of those having a normal weight. We did not find a linear association among deciles of BMI and hypertension in any of the groups studied. Instead, the association of BMI with hypertension might be quadratic and mainly seen in high extreme values. It seems that the influence of BMI on hypertension is only seen in adolescent women classified as overweight and obese, while increases of BMI in women classified as thin or normal do not seem to have any effect on hypertension.

Despite the fact that both countries had similar BMI values and distributions, Norwegian adolescent women were considerably taller than Argentinean ones. This difference in height may reflect the nutrition status of a long-term established developed country and a country in transition. The low rate of thinness in Argentina could indicate a nutritional improvement not yet reflected in the height of its population [15].

Both studies are regarded as fairly representative for their country as a whole. Young-HUNT studied all adolescents from the county of Nord-Trøndelag. In this county, higher education prevalence and average income are slightly lower than the average in Norway, while regarding geography, economy, industry, sources of income, age distribution, morbidity and mortality it is quite close to the average for Norway [7]. Participants in ENNyS were selected using a probabilistic complex sample design including different socio-economic levels from both large and small cities of all provinces of Argentina in order to represent the whole country [8].

The values for systolic and diastolic pre-hypertension and hypertension turned out to be quite different in the two countries; however, methodological differences in the measurements preclude us to make further comparisons. Also, differences in climate, number of measurements taken, measurements in different environments and different blood pressure measurement devices were described between the two countries. Most importantly, an automatic oscillometric device was used in the Norwegian study and a mercury sphygmomanometer in the Argentinean study. Higher systolic values have been reported using oscillometric as opposed to manual methods. Between 5 and 12 mmHg variation in mean systolic blood pressure values were reported using an automatic compared to a manual device [16]. A similar mean values difference (12.8 mmHg) was found in our study between the Argentinean and the Norwegian adolescent girls. This fact could explain the high rates of systolic hypertension observed in the Norwegian group and not in the diastolic one.

One limitation in this study is that the average of two measurements of blood pressure was used to define high blood pressure, whereas the standards used as reference required the average of three measurements. Other limitations are the lack of information on possible confounding factors such as physical activity and particularly the fact that data was collected between 1995 and 1997 in Norway and between 2004 and 2005 in Argentina.

In our opinion, the information shown in this article raises the need for further research in order to identify better the extent of obesity and hypertension in middle and high-income countries. Confirmation of these findings would imply major efforts to identify young population with risk of obesity and hypertension in order to design interventions to prevent the consequences of such entities on the quality of their current and future lives.

## Conclusion

This study confirms a current world health problem by showing high prevalence of obesity in adolescents and its association with hypertension in two different countries (one developed and one in transition).

## Abbreviations

BMI: Body Mass Index; HUNT: Norwegian Health Study of Nord-Trøndelag; ENNYS: Argentinean National Survey of Nutrition and Health; INDEC: Argentinean Institute of Statistics and Census

## Competing interests

The authors declare that they have no competing interests.

## Authors' contributions

JMB had the original idea. MSP, RMH, LG and GC compiled the databases and made the statistical analysis, and MSP, RMH and JMB wrote the manuscript in collaboration with LG, GC, TV and TLH. All authors have read and approved the final manuscript.

## Pre-publication history

The pre-publication history for this paper can be accessed here:


